# New approach to expedite the delivery of the final crowns for teeth requiring crown lengthening surgery: a pilot study

**DOI:** 10.1186/s12903-022-02491-w

**Published:** 2022-11-02

**Authors:** Se-Lim Oh, Luz Abrera-Crum, Ji Seung Yang, Seung Kee Choi

**Affiliations:** 1 Department of Advanced Oral Sciences and Therapeutics, School of Dentistry, University of Maryland, Baltimore, MD USA; 2 Department of General Dentistry, School of Dentistry, University of Maryland, Baltimore, MD USA; 3 Department of Human Development and Quantitative Methodology, College of Education, University of Maryland, College Park, MD USA

**Keywords:** Clinical crown lengthening, Lithium-disilicate crown, Crestal bone level, Intraoral imaging, Periodontics, Prosthodontics

## Abstract

**Background:**

The healing period from crown lengthening procedures (CLPs) often delays the final crown delivery. This study aimed to explore the feasibility of a new approach expediting the delivery of the final crowns for teeth requiring CLPs.

**Methods:**

Teeth requiring CLPs and single-crown restorations between the canine and the second molar were included. After the initial tooth preparation, a CLP was performed. In the experimental group, the final tooth preparation and final impression were made during the CLP; the final crown was then delivered at the suture-removal appointment. In the control group, the final impression was made 8 weeks after the CLP. The level of gingival margin (GM), pocket depth (PD), and crestal bone levels (CBLs) were compared between the two groups before CLPs (T0), at delivery of the crowns (T1), and at 12 months in function (T2).

**Results:**

Twenty-one lithium-disilicate crowns were delivered to 20 subjects and followed up. The mean interval between the CLPs and the delivery of crowns was 2.5 weeks for the experimental group and 12 weeks for the control group. No significant differences were observed between the two groups in the level of GM, PD, and CBLs at each time point. No significant treatment difference in crestal bone loss was observed between the two groups at T2 (Experimental = -0.11 mm, Control = -0.03 mm; *p =* 0.67).

**Conclusion:**

Making the final tooth preparation and the final impression at the CLP significantly reduced the time between the CLP and the delivery of the final crown and showed comparable clinical outcomes.

## Background

Periodontal tissues interact with dental restorations. Restorative procedures, such as crown preparation, gingival displacement during impression, and the long-term use of resin provisional crowns, can negatively affect the periodontium [1]. The location of restorative margins and the quality of restorations also impact periodontal health [2]. Therefore, in cases where caries extends below the gingival margin (GM), a crown lengthening procedure (CLP) is often required so that the crown margin would not violate the dentogingival junction (supracrestal tissue attachment) and the adequate final impression could be made [3]. While the apically positioned flap with osseous surgery is commonly performed to extend the clinical crown, the duration of the healing period from CLPs to initiating restorative procedures is controversial [4].

Postoperative 2–4 mm of gingival recess was reported between 6 weeks and 6 months [5], and the original supracrestal tissue attachment was re-established 3 months after the CLP [6, 7]. The crown lengths gained from CLPs were significantly decreased at 6 months due to the coronal migration of the GM, which was observed for 12 months following CLPs [8–10]. Therefore, a wide range from 6 weeks to 6 months is recommended for the healing period before making the final impression to fabricate definitive crowns after CLPs.[11].

Another controversial issue regarding CLPs is the amount of bone reduction [4]. Based on the concept of supracrestal tissue attachment [12], there is a necessary amount of root surface to be exposed to restore this dimension. However, a limitation of bone reduction also exists due to local anatomical factors, such as the furcation and crown-to-root ratio. Nearly 40% of the mandibular molars developed furcation involvement at 5 years after the CLPs when the distance from the furcation entrance to the margin of the temporary crown or excavated caries line was less than 4 mm [13].

Thus, there is little consensus on the postoperative changes following CLPs among studies. The optimal timing of restorative treatment has not been systemically investigated. Since packing gingival cords to make a final impression could injure the soft tissue still in healing and the tooth preparation was not definitive at the CLP, restorative treatment is generally initiated 6 weeks after CLPs [5, 6]. However, the long-term use of provisional restorations is not desirable. This waiting period delays the final crown delivery, which is often the patient’s chief complaint.

Technical developments have been made in restorative dentistry through digital workflows, such as computer-aided design/computer-aided manufacturing (CAD/CAM) and intraoral imaging. Studies have reported that digital impressions are equally or more accurate than those made with various conventional impression materials [14–16]. Since the intraoral imaging system allows us to obtain the impression without applying materials around the teeth, the final impression could be made during the CLP without using gingival cords and without increasing any postoperative complications. If the final tooth preparation and making the final impression are performed at the CLP, the delivery of a definitive crown can be expedited and the long-term use of the provisional crowns can be avoided.

The purpose of this randomized pilot clinical study was to explore the feasibility of the proposed approach, in which the final tooth preparation and final impression were made at the CLP and the crown was delivered at the following postoperative appointment. The conventional approach, in which restorative procedures began at 8 weeks after CLPs, was the control group. The study compared clinical and radiographic outcomes around lithium-disilicate crowns made from the two different restorative approaches to assess their impacts on periodontal health.

### Methods

### Ethical approval

The study was a pilot randomized controlled trial (RCT). The protocol for this study was approved by the institutional review board (IRB) at the University of Maryland, Baltimore (HP-00073913) and was registered in ClinicalTrials.gov (NCT03064217). The study was conducted from 2017 to 2021. The study reports followed the CONSORT guidelines for RCTs.

### Study design and data collection

**Fig. 1:**
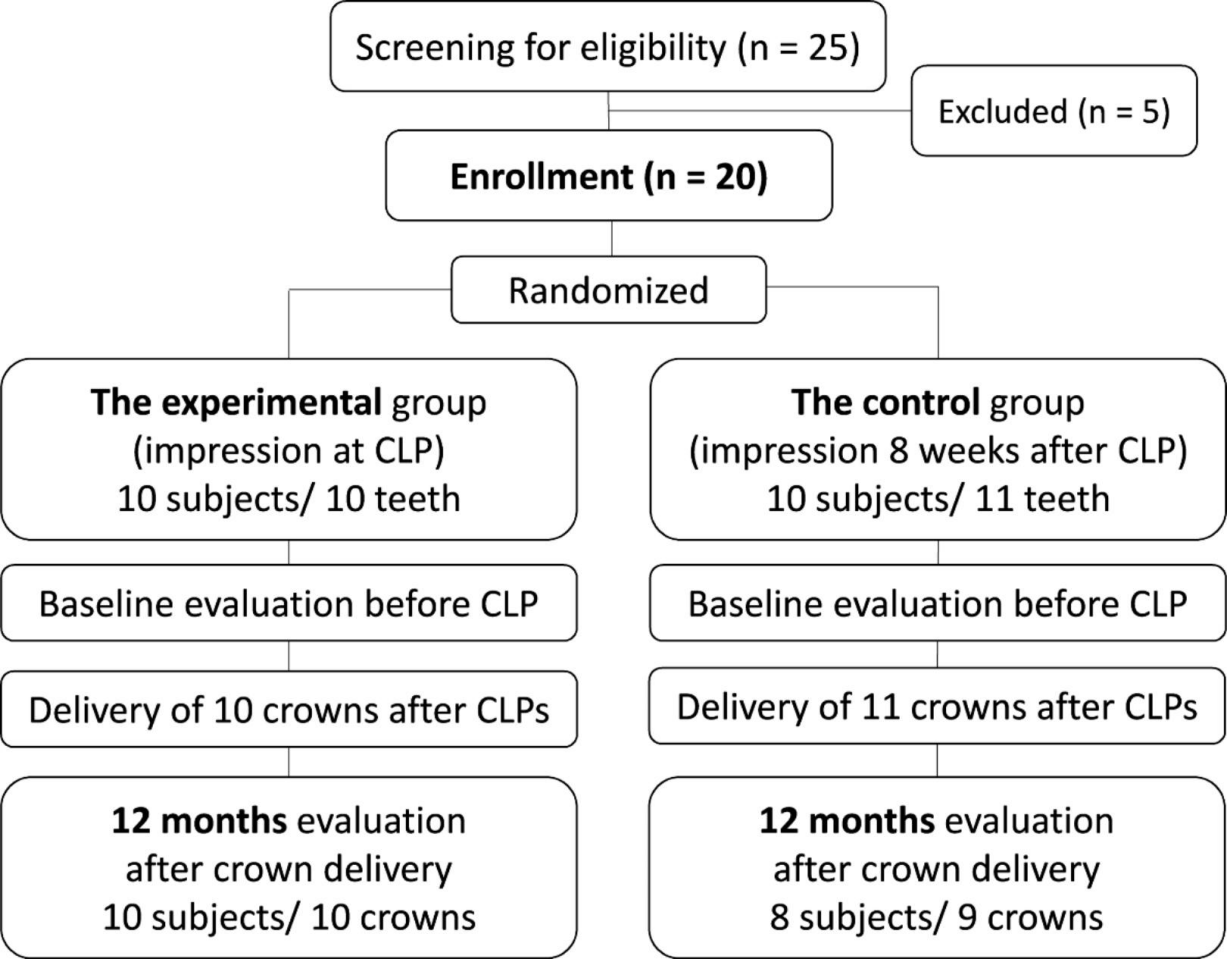
A flowchart of the study

This study recruited patients from dental clinics at the University of Maryland School of Dentistry. Subjects with the following conditions were included: (a) age > 18 years, (b) a non-splinted, single tooth-supported crown needed, (c) that tooth must be in the area between the canine and the second molar, and (d) a CLP is required prior to the fabrication of a lithium-disilicate crown due to caries extending below the gingival margin. Exclusion criteria were as follows: (a) uncontrolled hypertension, (b) HbA1c level of 6.5% or above, (c) history of the long-term corticosteroid use (> 6 months), (d) history of taking oral/IV bisphosphonates within the past 2 years, (e) history of anticoagulant use, and (f) smoker. Among the 25 patients screened, 20 subjects were enrolled after they signed the research informed consent form. The enrolled subjects were randomly assigned to either the experimental or the control group using a random number table by the principal investigator, Se-Lim Oh (SO). All CLPs were performed by SO. The study design is illustrated in Fig. 1.

**Fig. 2:**
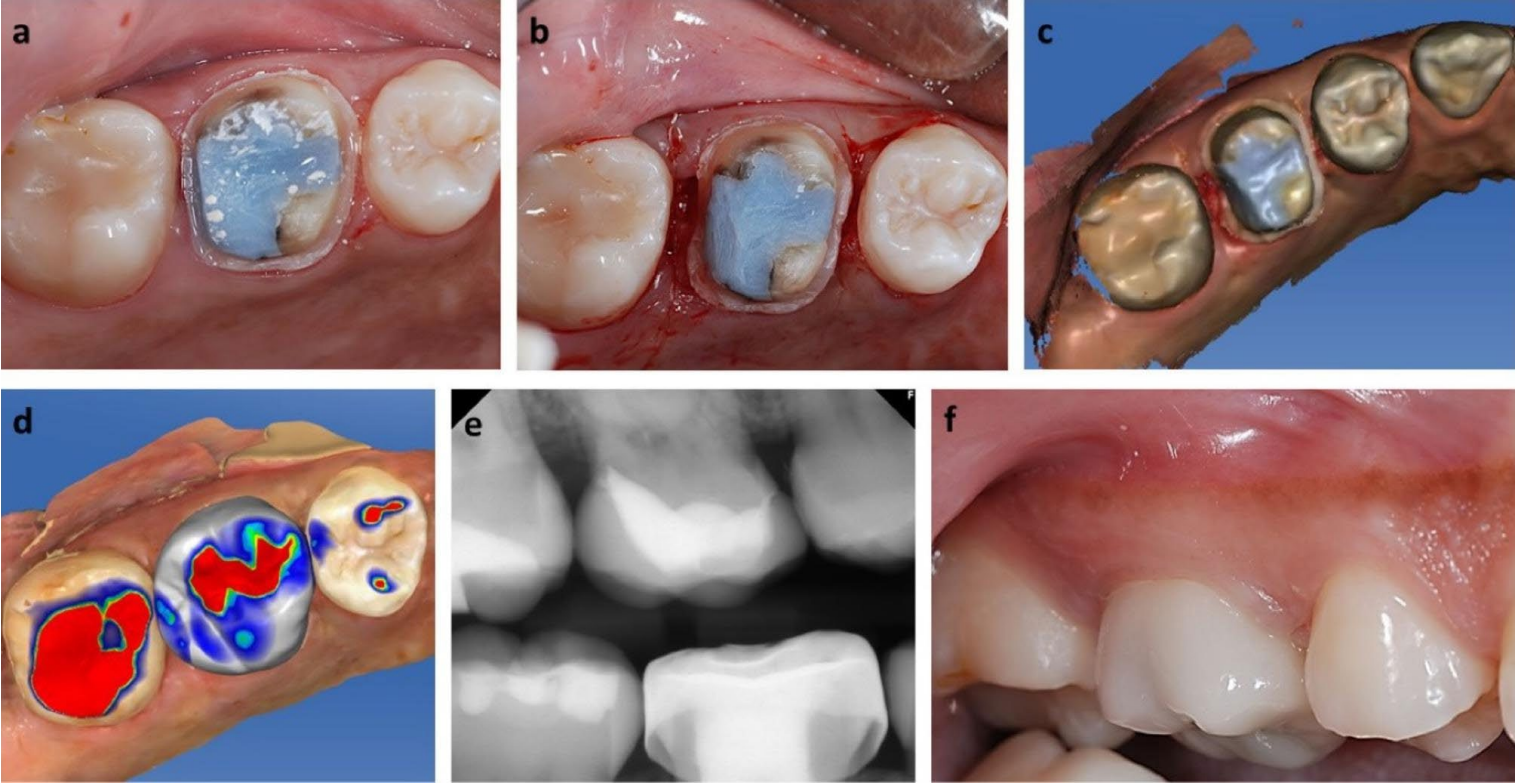
A workflow example from one of subjects in the experimental group. (a) Presentation after initial tooth preparation revealing the absence of axial tooth structure on the distal surface of #16. (b) Completion of tooth preparation and clinical crown lengthening. (c) Acquisition of intraoral scanning of #16 and its neighboring areas. (d) Computer-aided design (CAD) of the definitive restoration. (e) Radiograph of the definitive restoration at delivery (T1). (f) Presentation of the lithium-disilicate crown at 12 months (T2)

Figure 2 shows one of the cases in the experimental group. The maxillary right first molar exhibited deep caries on the distal surface and the initial tooth preparation margin was still on the core material (Fig. 2a). Therefore, a CLP was performed. A crevicular incision was made on the facial and lingual/palatal sides of the selected teeth area, and full thickness flaps were retracted. The tooth was reprepared by a restorative dentist (Luz Abrera-Crum) to place the final preparation margin on the sound tooth structure during the CLP. Consequently, the preparation margin became closer to the alveolar crest. Osseous reduction at the deep caries site was performed until the distance from the final preparation margin to the crestal bone was approximately 2 mm. Interrupted sutures were placed with 5 − 0 Monocryl undyed monofilament (Ethicon, Sommerville, NJ, USA) to close the flaps and control bleeding (Fig. 2b). The final impression was made using an intraoral scanner (Omnicam, Dentsply Sirona, Charlotte, NC, US; Fig. 2c) without using gingival retraction cords. A lithium-disilicate crown was fabricated using a CAD/CAM block (IPS e.max; Ivoclar Vivadent, Amherst, NY, US; Fig. 2d). Then, the fabricated lithium-disilicate crown was delivered 2–3 weeks after the surgery at the suture removal appointment (Fig. 2e).

In the control group, a CLP was performed after the initial tooth preparation. After retracting the full thickness flaps, osseous reduction was performed to expose approximately 2 mm of sound tooth surface above the alveolar crest at the deep caries site; sutures were placed to reposition the flaps. Eight weeks after the CLP, the tooth was reprepared and the final impression was made using the same intraoral scanner after packing the #00 gingival cord (Ultrapak, Ultradent South Jordan, UT, US). Then, a lithium-disilicate crown was fabricated with the same CAD/CAM block used for the experimental group and delivered. All lithium-disilicate crowns were luted using an adhesive luting composite (Variolink; Ivoclar Vivadent, Inc., Amherst, NY, US) following the manufacturer’s instructions.

No antibiotics were prescribed in conjunction with the CLPs. 0.12% Chlorhexidine Gluconate (3 M, St. Paul, MN, US) was prescribed for 7–10 days. Over the counter (OTC) analgesics (500 mg Tylenol or 200 mg ibuprofen) were offered to all subjects immediately after the surgery before the subjects were dismissed. All subjects were advised to take OTC analgesics as needed at home. To assess the subject pain level, the number of OTC analgesics taken at home was recorded at the postoperative follow-up appointment. After the delivery of the crowns, the subjects received other planned treatments while either oral prophylaxis or periodontal maintenance was performed on a regular basis until they completed their participations in the 12-month follow-up (Fig. 2f).

The level of GM and probing depths (PDs) were obtained from six sites per tooth as a part of comprehensive or periodic oral evaluation with a probe before the CLPs and at 12 months following the crown delivery; the measurements at the deep caries sites from the teeth were analyzed in this study. The level of GM was evaluated either from the caries margin (prior to CLPs) or the crown margin to free GM (after the delivery of the crown); positive numbers indicate gingival recession, and negative numbers indicate gingival overgrowth. The PD was measured from the GM to the base of the pocket.

**Fig. 3:**
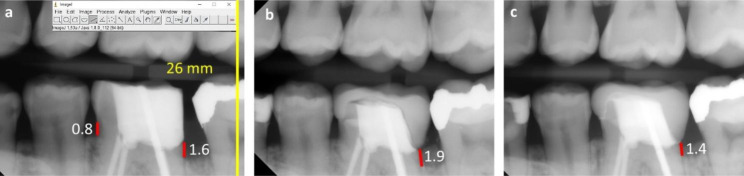
Example of crestal bone level measurements (mm) using ImageJ program. (a) A radiograph was opened in the ImageJ program. The known length (26 mm) of the sensor was used to calibrate the image. Crestal bone level measurements on the deep caries and non-caries sides before the CLP. (b) Crestal bone level measurement on the caries side at crown delivery. (c) Crestal bone level measurement on the caries side at the 12-month follow-up

Bitewing radiographs were used to measure crestal bone levels (CBLs) around the teeth. To obtain images with a minimum distortion, an extension cone paralleling (XCP) film positioning device (Rinn XCP alignment system; Dentsply Sirona, Charlotte, NC, US) was used to place a radiographic sensor as the target tooth was centered and the x-ray tube head was perpendicular to the sensor. A blinded evaluator, Seung Kee Choi (SC), measured crestal bone level (CBL) for all subjects using ImageJ [17] before CLPs (T0), at the delivery of crowns (T1), and at the 12 months follow-up (T2). Figure 3 presents examples of measurements in one subject from the experimental group. The known intraoral X-ray sensor lengths (26 mm × 36 mm) were used to calibrate each image (Fig. 3a). The CBL was measured from the most coronal tooth structure on the deep caries side to the coronal aspect of the alveolar crest at T0 (Fig. 3a). After inserting the crowns, the CBL was measured from the crown margin to the coronal aspect of the alveolar crest on the previous caries side at T1 and T2 (Fig. 3b–c). Crestal bone loss was calculated by subtracting CBL at T1 from T2 (T2-T1) with a positive value indicating bone loss.

### Statistical analysis

Calibration for the blind evaluator (SC) was performed by SO; SC and SO independently measured CBLs on radiographs of 10 teeth. The Pearson correlation coefficient between the two evaluators was 0.85, indicating a high positive correlation. The measurements from the blind evaluator were used for the statistical analysis to minimize bias.

This study is the first clinical trial to determine the feasibility and safety of the proposed approach. Therefore, 10 subjects in each group were selected as a pilot study to clarify whether making the final impression at the surgery is acceptable or not [18]. To test the null hypothesis of no difference in the level of GM, PDs and CBLs at T0, T1, and T2 between the experimental and control groups, independent t-tests were conducted. The confidence interval (CI) of the treatment difference in crestal bone loss between the two groups was evaluated against the non-inferiority margin (0.5 mm), which was determined based on the study by Fickl et al. [19] and clinical judgement.

The current sample size allowed detection of a large effect size of Cohen’s d = 0.8 with 0.53 and 0.7 power when the one-tail Type I error rate is 0.05 and 0.1, respectively. When the mean of crestal bone loss in each group was compared against zero (gold standard) using a one sample t-test, the power reached 0.8 for a large effect size of Cohen’s d = 0.8 if the one-tail Type I error rate was 0.05. Overall, the study sample size might be underpowered but allowed us to detect at least a large effect size if there were any. Data analysis was performed with GraphPad Prism (version 9; GraphPad Software, San Diego, CA, US); *p < 0.05* was considered significant.

## Results

Table 1 summarizes the patient and tooth characteristics of this study at baseline. A total of 20 subjects (14 women and 6 men) participated in this study. Their mean age was 51 ± 20 years (range, 19–79 years) at the time of the CLPs. The caries was extended approximately 1 mm below the mesiofacial (1 case), distofacial (2 cases), mesial (5 cases) or distal (13 cases) GMs. The mean PD at the deep caries site was 2.7 ± 0.9 mm. With respect to the CBL measurements, the mean distance from the core margin to the crestal bone at the deep caries site was 1.6 ± 0.7 mm; the mean distance from the CEJ to the crestal bone at the non-caries site was 1.9 ± 0.7 mm at T0.

**Table 1:** Patient and tooth characteristics at clinical crown lengthening procedures (T0)

**Patient characteristics at T0**				
	Experimental	Control	Total	*p value*
*Age* (mean ± SD)	55.8 ± 22.0	45.5 ± 16.6	50.6 ± 19.7	*0.25* ^*^
*Gender (the number of subjects)*				*0.33* ^*†*^
male	4	2	6	
female	6	8	14	
*Postoperative analgesics (the number of subjects)*				*0.03* ^*†*^
0 tablet 1 tablet	8 (80%) 2 (20%)	3 (30%) 7 (70%)	11 (55%) 9 (45%)	
*At home analgesics in total (the number of subjects)*				*0.21* ^*†*^
0 tablet	7 (70%)	4 (40%)	11 (55%)	
1 tablet	1 (10%)	5 (50%)	6 (30%)	
2 tablets	1 (10%)	0	1 (5%)	
3 tablets	1 (10%)	1(10%)	2 (10%)	
**Tooth characteristics at T0**				
At deep caries site (mean ± SD; mm)	Experimental (n1 = 10)	Control (n2 = 11)	Total (N = 21)	*p value*
*Gingival recession*	-1.1 ± 0.7	-1.1 ± 0.5	-1.1 ± 0.6	*0.97* ^*^
*Pocket depth*	2.8 ± 0.9	2.5 ± 0.9	2.7 ± 0.9	*0.54* ^*^
*Crestal bone level*	1.78 ± 0.8	1.41 ± 0.7	1.6 ± 0.7	*0.24* ^*^

SD = standard deviation; ^*^ independent t-test; ^†^ chi-square test

Two subjects in the experimental group and seven subjects in the control group took one tablet of analgesics (either 500 mg Tylenol or 200 mg ibuprofen) immediately after completion of the CLPs. While significantly more subjects in the control group took analgesics after CLPs (chi-square, *p = 0.025*), there was no difference in the administration of at-home analgesics between the two groups; seven subjects in the experimental group and four subjects in the control group did not take analgesics at home (chi-square, *p = 0.21*).

Twenty-one lithium-disilicate crowns were delivered to 20 subjects after CLP. The mean interval between CLPs and the delivery of crowns was 2.5 weeks for the experimental group and 12 weeks for the control group. At the delivery of the final crowns, the mean CBL measurement was 1.9 ± 0.8 mm from the crown margin to the crestal bone at the previous deep caries site (Table 2). No discomfort was reported in any of the subjects after the delivery of the final crowns.

**Table 2:** Clinical and radiographic measurements around the crowns at crown delivery (T1) and the 12-month follow-up (T2). The amount of crestal bone loss was calculated by subtracting CBL measurements at T1 from T2 (T2-T1) with a positive value indicating bone loss

**Tooth characteristics at T1**				
At deep caries site (mean ± SD; mm)	Experimental (n1 = 10)	Control (n2 = 11)	Total (N = 21)	*p value*
*Crestal bone level*	1.85 ± 1.0	1.94 ± 0.7	1.9 ± 0.8	*0.83* ^*^
**Tooth characteristics at T2**				
At deep caries site (mean ± SD; mm)	Experimental (n1 = 10)	Control (n2 = 9)	Total (N = 19)	*p value*
*Gingival recession*	-0.4 ± 0.8	-0.4 ± 0.9	-0.4 ± 0.8	*0.91* ^*^
*Pocket depth*	2.7 ± 1.2	2.4 ± 1.0	2.6 ± 1.1	*0.62* ^*^
*Crestal bone level*	1.74 ± 1.1	1.91 ± 0.6	1.8 ± 0.9	*0.68* ^*^
*The amount of crestal bone loss*	-0.11 ± 0.4	-0.03 ± 0.5	-0.1 ± 0.4	*0.67* ^*^

SD = standard deviation; ^*^ independent t-test

Two subjects in the control group did not make the 12 months follow-up. Nineteen crowns (10 in the experimental and 9 in the control group) from 18 subjects were followed up for 12 months (Table 2). No outstanding issues, such as recurrent caries, clinically detectable gingival inflammation, and fracture/dislodging of the crowns, were observed in the 19 crowns. The mean distance from the GM to the crown margin was -0.4 ± 0.8 mm, the mean PD was 2.6 ± 1 mm, and the mean CBL was 1.8 ± 0.9 mm at the previous deep caries site at T2. There were no significant differences in the level of GM, PD, and CBL between the two groups (independent t-test; *p = 0.91, p = 0.62*, and *p = 0.68*, respectively).

**Fig. 4:**
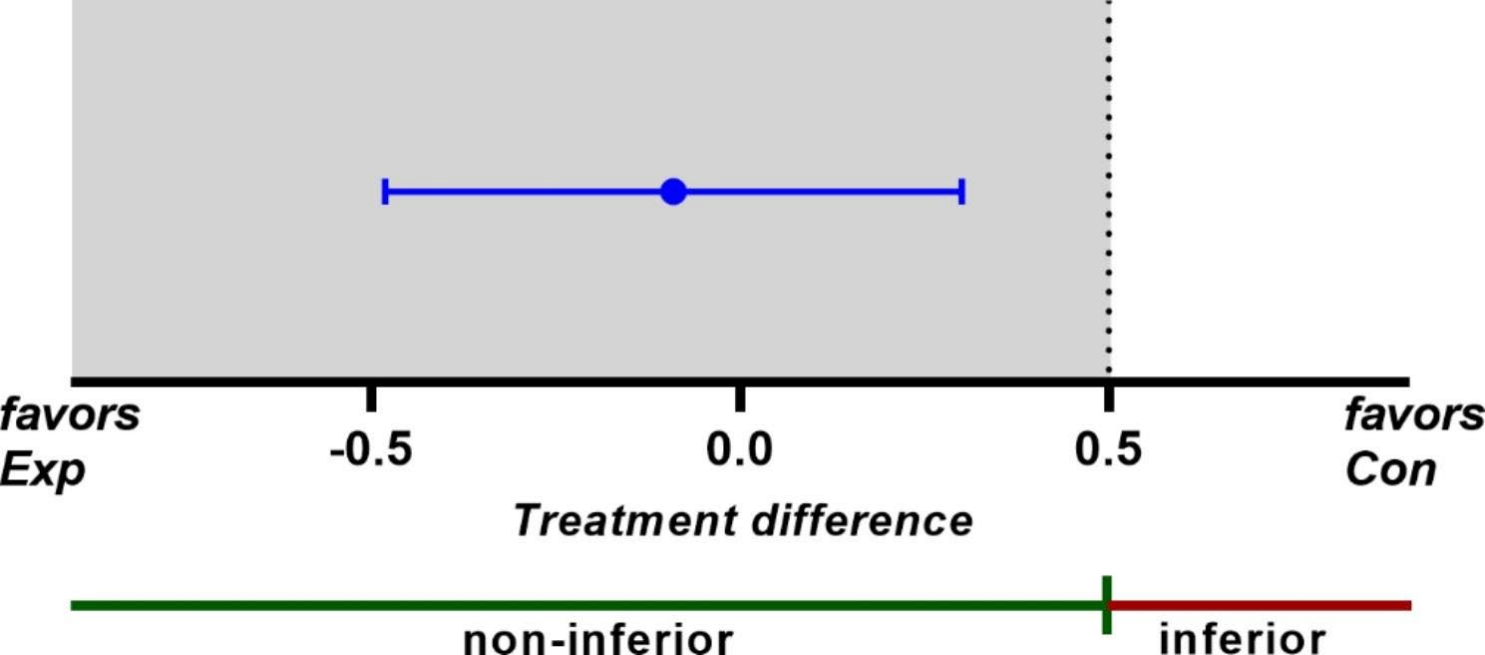
Test for the non-inferiority on the deep caries side. Confidence interval (CI) of the treatment difference and the non-inferiority margin (0.5 mm) are shown. The 95% CI of the treatment difference [-0.47, 0.31] is entirely covered by the non-interiority zone

The amount of crestal bone loss (mm) from T1 to T2 between the two groups was compared. In both groups, the means of crestal bone loss at the previous deep caries site were not significantly different from zero (95% confidence interval (CI) = [-0.37, 0.15], t_df=9_ = -0.95, *p = 0.37* for the experimental group; 95% CI = [-0.37, 0.32], t_df=8_ = -0.17, *p = 0.86* for the control group). There was also no significant difference in the mean crestal bone loss at the previous deep caries site between the two groups (Table 2; independent t-test, *p = 0.67*). The 95% CI of the treatment difference in crestal bone loss at the previous deep caries site was from -0.47 to 0.31, which entirely lies within the non-inferiority zone (Fig. 4).

## Discussion

Interproximal deep caries and associated gingivitis hamper the fabrication of adequate restorations. Although deep-margin elevation approach may be applied to avoid CLPs [20], in this study, CLPs were performed to avoid the violation of supracrestal tissue attachment and to place the final crown margin on the sound tooth structure based on the conventional restorative viewpoint [5].

Considering the prevalence of caries [21], CLPs are among the most frequently performed periodontal surgeries. However, most studies related to CLPs have reported the positions of GM and CBL before CLPs using custom stents or different reference points [4]. One study reported mean distances from the reference stent to the GM and the alveolar crest were 4.6 and 9.3 mm before CLPs [6]; it is difficult for clinicians to visualize how far the caries extended below the GM. In this study, the teeth exhibited deep interproximal caries extending 1 mm below the GM and 1.6 mm close to the crestal bone. The mean PD at the deep caries site was 2.7 mm (Table 1). Therefore, it is sensible for clinicians to expect a possibility of CLP under such conditions and to discuss with patients in order to fabricate an adequate full-coverage crown.

Studies have suggested up to 1–5 mm of bone reduction with respect to the anticipated restorative margin based on the predetermined dimension of supracrestal tissue attachment [5, 6, 8]. In this study, the amount of bone reduction was determined after the final crown margin was established for the experimental group. Although the exact amount of bone reduction was not measured in this study, the surgeon (SO) was able to see the final restorative margin during the CLPs and removed the crestal bone until the distance from the final crown margin to the crestal bone was approximately 2 mm.

The goal of CLPs is to deliver definitive crowns. However, limited information on the final restorations following CLPs is available; most studies have focused on the changes either in the level of GM or alveolar bone height following CLPs [11]. While no studies have reported the types of final crowns, lithium-disilicate crowns were delivered to all subjects in this study because of their strength and biocompatibility, such as less plaque accumulation and little inflammatory reactions from the soft tissue [22, 23].

The primary providers for the subjects in this study were dental students and their chair times are considerably long even for a simple procedure because of their learning curve. Therefore, we did not attempt the same day delivery of final crowns during the surgical visit, which might be possible with highly trained clinicians. The proposed approach allowed the delivery of final crowns within 3 weeks from the CLPs. When we compared the clinical and radiographic outcomes between the experimental and control groups, the concept of non-inferiority was applied because the superiority of the proposed approach was not necessarily presumed. It was more reasonable to investigate whether our approach achieved similar results to the conventional approach [24].

Studies have reported that intraoral radiographs made with an intraoral paralleling technique with alignment systems allow accurate images of crestal bone in relation to the root to be obtained [25, 26]. While adding a bite registration to an alignment system may improve the repeatability of image producing [27], fabricating individual jigs to take radiographs is not feasible in routine practice. Clinicians use radiographs obtained with an alignment system to compare CBLs. The cut-off for significant change in radiographs taken routinely with a positioning alignment system is 0.5 mm [26]. Therefore, in this study, the treatment difference margin for non-inferiority between the two groups was set at 0.5. Our results demonstrated the non-inferiority of the proposed approach with 95% confidence (Fig. 4).

While the proposed approach achieved similar outcomes to the conventional approach, there were a few setbacks for the proposed approach. It was difficult to anticipate the final position of the facial GM from the flap positioning, although the final crown margin at the deep caries site was slightly below the GM at the 12-month follow-up. Therefore, the proposed approach may be utilized in areas with less esthetic concerns. Occasionally, isolation from the hemorrhage was difficult for scanning. The foundational core and provisional restorations should be made with optimal quality to minimize gingival inflammation before CLPs. This study did not assess gingival inflammation via either bleeding on probing or measuring inflammatory cytokines as objective measures [28]. Thus, measures for gingival inflammation should be included in future clinical studies.

## Conclusion

The proposed approach, in which making the final impression at CLP, is a feasible option to expedite the delivery of the final crowns for teeth requiring CLPs, especially in the non-esthetic zone. To confirm the effectiveness of the proposed approach, prospective studies with the long-term follow-up in a large sample size using robust statistical analysis are desired. Future clinical trials need to be conducted under routine clinical settings, including a wide range of patient populations, different types of final restorations, and clinicians with various levels of experience.

## Data Availability

The datasets used and analyzed in this study are available from the corresponding author upon reasonable request.

## Abbreviations

CLP: clinical crown lengthening
PD: pocket depth
GM: gingival margin
CBL: crestal bone level
